# The complete plastid genome of *Ficus erecta* (Moraceae)

**DOI:** 10.1080/23802359.2020.1768914

**Published:** 2020-09-21

**Authors:** Yanhong Wang, Yanhong Cui

**Affiliations:** a College of Horticulture and Landscape Architecture, Heilongjiang Bayi Agricultural University, Heilongjiang, China; b Center of Laboratory Equipment Management, Heilongjiang Bayi Agricultural University, Heilongjiang, China

**Keywords:** Plastid genome, *Ficus erecta*, phylogeny, Moraceae

## Abstract

*Ficus erecta* is a wild relative of *F. carica,* which is an important economic plant. Here, we determined the complete plastid genome of *F. erecta* using the Illumina paired reads to provide genomic feature resources. The whole plastid genome of *F. erecta* is 160,603 bp in length, containing two inverted repeats (IRs) of 25,899 bp separated by a large single-copy (LSC 88,640 bp) region and a small single-copy (SSC 20,165 bp) region. The complete plastome sequence of *Ficus erecta* will provide a useful resource for the evolutionary biology study as well as for the phylogenetic studies.

The genus *Ficus* includes about 735 species with a selection of syconiumsis, an extraordinary genus to investigate symbiotic relationships between flowers and their pollinators (Berg and Corner 2005, Galil 1973). Figs are very important economic plants as food and medicinal resources. Besides, figs also play important roles in ecology system in some regions. *Ficus carica* is one of the oldest cultivated plant for food resources but always susceptible to some fungus diseases such as *Ceratocystis* canker which is transmitted through soil (Kajitani and Masuya 2011). *Ficus erecta* is a wild relative of *F*.* carica* with resistance to *Ceratocystis* canker, provide a feasible way to combat soil-transmitted diseases. The genome of male *F*.* erecta* was assembled with high quality and provided insights into *Ceratocystis* canker resistance breeding strategies and the mechanisms of *F*.* erecta* resistance to diseases.

In this study, we downloaded total 3.9 G data of high-quality reads (NCBI accession # DRR187744) using the fastqdump software, which was released by the *F*.* erecta* genome sequencing project (Shirasawa et al. 2019). The individual for sequencing was a male *F*. *erecta* tree “FE-Hiroshima-1” which was grown naturally in Higashi-Hiroshima city in Japan. The sample was stored at Kazusa DNA Research Institute herbarium, Japan.

Sequenced reads were then used to assemble the complete plastid genome using NOVOPlasty with a rpoA CDS (NC_028185) as a seed sequence (Dierckxsens et al. 2017]). We performed the annotation using Geneious 10.2.2 and adjusted the protein-coding genes manually in order to make sure all of these genes were maintained as open reading frames. IR boundaries for the draft plastome were confirmed by BLAST. The physical map of the plastid genome was conducted via OGDraw online (http://ogdraw.mpimp-golm.mpg.de/) (Lohse et al. 2013). Finally, we obtained a chloroplast genome of *F. erecta* and submitted the whole genome to GenBank (MT093220).

The plastome of *F. erecta* is a double-stranded circular DNA of 160,603 bp in length with the typical quadripartite structure of angiosperms, containing two inverted repeats (IRs) of 25,899 bp separated by a large single-copy (LSC) region and a small single-copy (SSC)region of 88,640 and 20,165 bp, respectively. The plastid genome of *F. erecta* contains 112 genes, including 78 protein-coding genes, 4 ribosomal RNA genes, and 30 transfer RNA genes. The overall GC content of *F. erecta* plastid genome is 35.9% and the corresponding values in LSC, SSC, and IR regions are 33.5, 28.9, and 42.6%, respectively.

We also used the protein-coding genes of *F. erecta* and another 9 Moraceae species to reconstruct a maximum likelihood tree through RaxML (Stamatakis 2014) under the GTRGAMMA substitution model, with 1000 bootstraps on CIPRESwebsite (Miller et al. 2010). The result shows that *Ficus* formed the clade corresponding to Ficeae. (Figure 1). The Moreae comprised a paraphyletic grade which was consistent with the previous study (Datwyler and Weiblen 2004).

**Figure 1.**
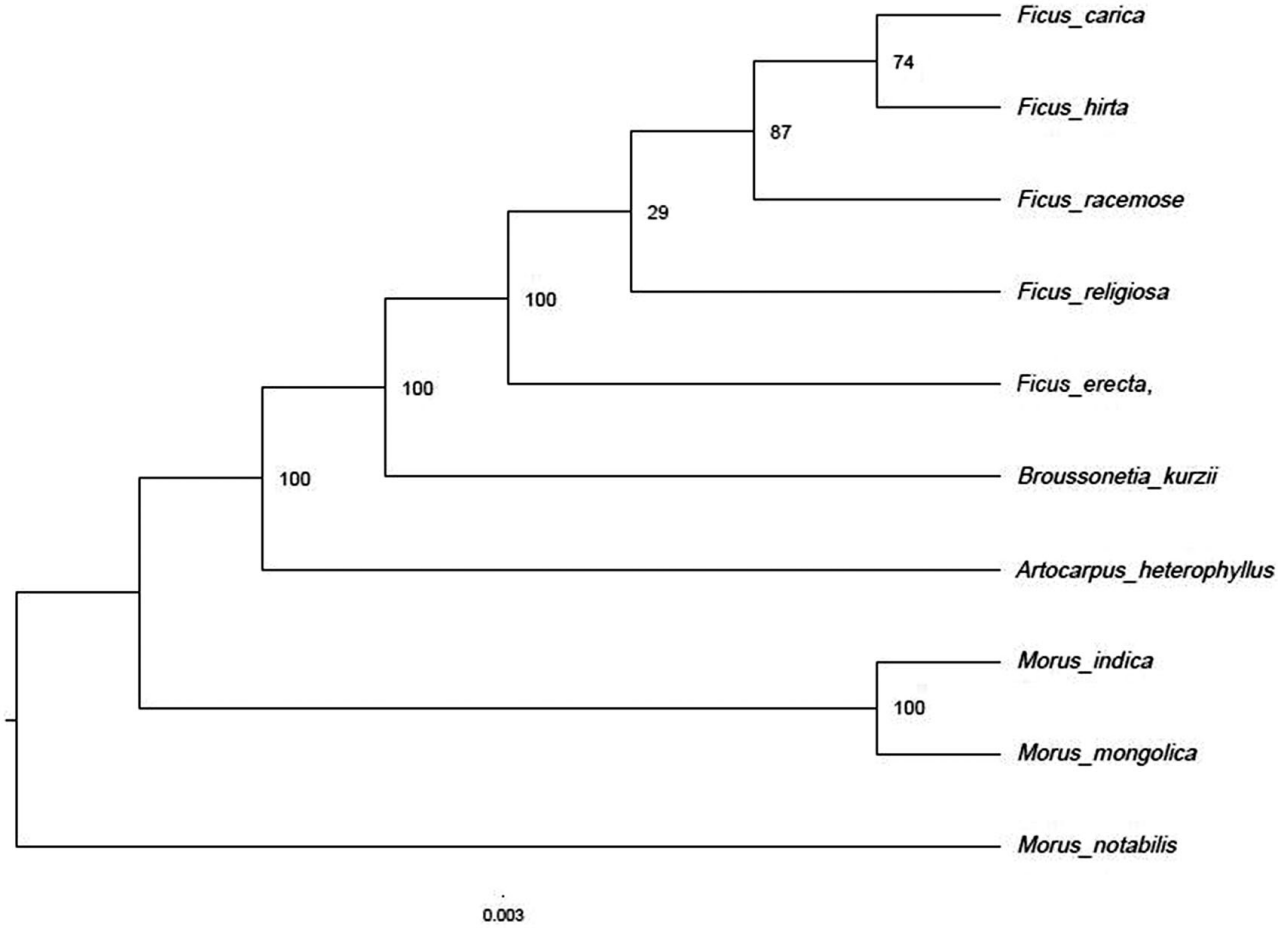
Phylogenetic tree (maximum likelihood) based on the protein-coding genes of 10 species. Numbers above branch aremaximum parsimony bootstrap percentages.These accession numbers are as follows:*Morusindica* (DQ226511), *Morusmongolica* (KM491711), *Morusnotabilis* (MK211167), *Ficus carica* (NC_035237), *Ficus hirta* (MN364706), *Ficus racemose* (NC_028185), *Ficus religiosa* (NC_033979), *Broussonetiakurzii* (NC_041637), *Artocarpusheterophyllus* (MK303549), *Antiaristoxicaria* (NC_042884).

## Data Availability

The data that support the results of this study are openly available in GeneBank (https://www.ncbi.nlm.nih.gov/genbank/) under the accession numbers MT093220 after the paper has been accepted. These raw data used in this study were derived from the following resources available in DNA Data Bank of Japan (DDBJ) Sequence Read Archive (DRA).
